# The impact of COVID-19 on parents from Black ethnic backgrounds in the UK: what we have learned and why it still matters

**DOI:** 10.1192/bjo.2025.10049

**Published:** 2025-07-08

**Authors:** Valentina Cardi, Valentina Meregalli, Chiara Tosi, Laura Sudulich, Juliana Onwumere

**Affiliations:** Department of General Psychology, University of Padova, Italy; Department of Psychological Medicine, Institute of Psychiatry, Psychology and Neuroscience, King’s College London, UK; Department of Neurosciences, University of Padova, Italy; Padova Neuroscience Centre, University of Padova, Italy; Department of Government, University of Essex, UK; Department of Psychology, Institute of Psychiatry, Psychology and Neuroscience, King’s College London, UK; National Institute for Health Research, Maudsley Biomedical Research Centre, South London and Maudsley National Health Service Foundation Trust, London, UK

**Keywords:** Parents, Black ethnic background, burden, COVID-19, minorities

## Abstract

**Background:**

People from ethnic minority groups are more likely to be impacted by global disasters than White ethnic groups due to pre-existing vulnerabilities. A lack of trust in mainstream support services, which have often accounted poorly for the needs of those communities, contributes to further discrimination and disadvantage.

**Aims:**

This study was conducted in 2022, soon after the COVID-19 pandemic, to survey the overall well-being and healthcare needs of UK families with a Black ethnic background.

**Method:**

A total of 2124 parents completed an online survey that included measures of psychological well-being, children’s difficulties, family healthcare needs and perception of support both before and after the COVID-19 pandemic.

**Results:**

Seventy per cent of parents reported high levels of stress, depression and anxiety, and over half identified high emotional and relational difficulties in their children. Higher levels of distress in parents correlated with greater difficulties in children and poorer parent–child relationships. Community support was associated with greater parental well-being and fewer child difficulties. Parents sought support from formal support networks when health issues were perceived as more severe.

**Conclusions:**

This study engaged a large sample of families from Black ethnic backgrounds, but recruitment may have been biased by sociodemographic characteristics. Levels of psychological distress were high, possibly due to pre-existing and enduring exposure to difficult life circumstances. Support from community networks was perceived as helpful, especially by those with milder levels of psychological distress. The strong association between parents’ and children’s well-being suggests that family-focused interventions could be beneficial, especially if culturally adapted.

Large-scale collective stressors, such as natural or human-caused disasters, pose significant threats to individuals, families and society at large.^1^ Emotional reactions to these events vary widely because they are influenced by several individual and environmental factors. On the one hand, personal, interpersonal and community resources can buffer psychological and emotional difficulties and promote post-traumatic growth.^2^ Conversely, pre-existing mental or social vulnerabilities, combined with the nature of the disaster (e.g. type or level of exposure, extent of life threat, duration), increase the risk of developing behavioural changes or trauma-related psychological conditions, such as acute stress disorder, post-traumatic stress disorder or depression.^2^

The COVID-19 pandemic has determined a global health crisis, with a significant negative impact on the general population.^3,4^ Increased rates of emotional distress and behavioural problems have been documented worldwide, especially among children and adolescents, who have suffered disruptions due to social isolation, loneliness, financial uncertainty and school closures.^5–7^ Parental psychological distress has also been associated with children’s poorer well-being,^8,9^ corroborating the evidence that parents’ and children’s responses to adverse events are highly inter-related.^8,9^

People from ethnic minority groups have been disproportionately impacted by the pandemic.^5^ These groups have faced higher risks of infection and more severe clinical outcomes,^5–8^ including higher rates of hospitalisation and mortality, but also poorer mental health outcomes (e.g. higher levels of depression, anxiety and stress)^9–12^ compared with White communities.^7,8,13^ In the UK, a comprehensive report on families from a Black ethnic background has documented the ‘multidimensional’ impact of the pandemic in the context of pre-existing inequalities and psychosocial adversities.^7^ Young people reported high levels of anxiety due to financial difficulties in the family and concerns about isolation from friends and school, exacerbated by the lack of access to the internet and digital tools. Parents reported challenges due to financial pressure, tension in the family, digital divide and unfamiliarity with the education system, as well as experiences of racism and discrimination within support services.^7^ This adds to the broad literature on obstacles to care in minority groups, mainly due to intersecting inequalities, shame, stigma, lack of trust, poor fit of the available services to one’s specific needs^7,14,15^ and longer waiting times for treatment compared with White British people.^15^

In 2021, our research group received funding from the UKRI Economic and Social Research Council (ESRC) to survey the well-being and healthcare needs of families from a Black ethnic background (i.e. African, Caribbean and related mixed ethnicities) in the UK. Parents reported on their own psychological well-being and symptoms of anxiety, depression and stress, and their children’s difficulties. They rated the extent to which the COVID-19 pandemic had impacted on the ability to seek support for their own or their children’s difficulties, and on the level of support perceived from services and the community.

## Method

### Participants

Participants were recruited from March to July 2022. Eligibility criteria were: being the parent of a child aged 6–24 years; having a Black ethnic background (e.g. African or Caribbean heritage, and mixed Black ethnic backgrounds); sufficient understanding of the English language; and access to a mobile device. Those who self-identified as presenting these characteristics were included in the study. To ensure wide reach, recruitment was advertised to several community- and faith-based organisations, mental health charities and ethnic minority associations across the UK. It was also advertised in primary and secondary mental healthcare clinical services and educational institutions that had collaborated previously with the team on similar projects. All procedures contributing to this work complied with the ethical standards of the relevant national and institutional committees on human experimentation, and with the Helsinki Declaration of 1975, as revised in 2013. All procedures were approved by the ethical committee at King’s College London, UK (protocol no. HR/DP-21/22-26510).

### Procedure

Participants provided written informed consent prior to completing an online survey on the Qualtrics XM platform (Provo, Utah, USA). They reported sociodemographic information about age, gender described at birth, ethnicity, relationship status, employment status, accommodation (e.g. owned/rented house), home facilities (e.g. number of rooms, garden), number of children they were caring for, caregiver status (primary caregiver, secondary caregiver), physical health problems and mental health problems. Participants were then asked to focus on one of their children, specifically the one they felt most worried about, and answered questions regarding the child’s age, education/employment status, physical health problems and mental health problems. They completed the Depression, Anxiety and Stress Scale (DASS-21^16^), the Warwick-Edinburgh Mental Well-being Scale (WEMWBS^17^), the Strength and Difficulties Questionnaire (SDQ^18,19^) and a questionnaire developed specifically by the study team for assessment of the impact of the pandemic on the family.

### Measures

#### DASS-21

The DASS-21^16^ is a 21-item, self-report questionnaire assessing the symptoms of depression, anxiety and stress (e.g. nervous tension, difficulty relaxing and irritability) experienced over the previous week. Each item is rated on a scale from 0 (‘did not apply to me at all’) to 3 (‘applied to me very much or most of the time’). For each of the three subscales, a score is calculated by multiplying by 2 the sum of the individual items; therefore, each subscale has a final score ranging from 0 to 42. Higher scores indicate greater symptom severity. Specific cut-offs are described for each subscale.^20^

#### WEMWBS

The WEMWBS^17^ is a 14-item, self-report questionnaire assessing mental well-being in the previous 2 weeks. Each item is rated on a scale from 1 (‘none of the time’) to 5 (‘all of the time’). The total score is calculated by summing the individual items, and ranges from 14 to 70. Higher scores are indicative of greater well-being (see https://warwick.ac.uk/fac/sci/med/research/platform/wemwbs/using/howto/ for cut-off scores).

#### SDQ

The SDQ^18,19^ is a 25-item questionnaire assessing behavioural and emotional difficulties in children and young people. In this study, the parent version was used (downloaded from the following website: https://www.sdqinfo.org/a0.html). Parents were asked to focus on the child they felt most worried about, and to answer the questions based on their child’s behaviour over the previous month. Each item is rated on a scale from 0 (‘not true’) to 2 (‘certainly true’). The questionnaire comprises five subscales assessing emotional symptoms, conduct problems, hyperactivity/inattention, peer relationship problems and prosocial behaviour. In the present study, the global difficulties score was considered and calculated by summing the scores of the first four subscales; the global difficulties score ranges from 0 to 40. Higher scores are indicative of greater difficulties (cut-off scores are reported in the original article^18^).

#### Impact of COVID-19 on parents and children

This survey was specifically developed by the study team to assess the impact of COVID-19 on the respondents and their children (the survey is available in Supplementary Materials). Participants rated the impact of the pandemic on their mental and physical health and their child’s well-being (including mental and physical health and social relationships). They reported on support sought from services (for themselves or their child), perceived quality of the parent–child relationship and level of parental exhaustion. They also rated the extent to which they felt supported by family, friends, members of the community, the child’s school (when applicable) and their general practitioner (GP), prior to and after the pandemic.

### Statistical analyses

Statistical analyses were performed by a social scientist (L.S., one of the co-authors). Descriptive data (e.g. mean, percentages) were used to summarise the demographic characteristics of the respondents and their children. The mean scores of the DASS-21, WEMWBS and SDQ scales, and the percentages of participants scoring in the severity ranges, were identified. Descriptive statistics (mean, median, percentages) were used to describe the impact of COVID-19 on parents and children, and paired sample *t*-tests assessed differences in perceived support pre- to post-pandemic. Two sets of linear regressions models were calculated, the first to establish whether parents’ physical and psychological health were associated with the child’s difficulties (SDQ global score) and the likelihood of seeking support for the child’s mental health during the pandemic. The selected predictors were: DASS-21 anxiety, stress and depression scores; WEMWBS total score; levels of parent physical exhaustion and mental exhaustion (self-reported in the COVID-19 survey, items PR1 and PR2); and the presence of a mental and/or physical illness in the parent and/or child.

The second set of regression models explored the relationship between COVID-19 and parents’ and children’s well-being. The dependent variables included DASS-21 anxiety, depression and stress scores, WEMWBS total score and SDQ global difficulties score. Predictors included perceived support since the onset of COVID-19 from family, school, friends, community and their GP, as well as the perceived negative impact of COVID-19 on the ability to seek support from others, the child’s social relationships and the parent–child relationship.

## Results

### Demographic characteristics

Participants’ demographic characteristics are reported in Table 1. A total of 2124 responses were collected, with approximately one half from male respondents (*n* = 1067, 50.24%). Participants’ age ranged from 20 to 65 years (mean 36.77, s.d. = 5.84). Most were married or in a civil partnership (*n* = 1948, 91.72%), in full-time employment (*n* = 1566, 73.73%) and owned the house where they were living in, at the time of participation (*n* = 1530, 72.03%). Most participants had a Black African ethnic background (*n* = 1304, 61.39%), 14.27% a Caribbean background and nearly a quarter a mixed background. A small proportion reported having a physical (*n* = 229, 10.80%) or mental health condition (*n* = 222, 10.47%). As per inclusion criteria, all were parents and almost 90% performed the primary caregiving role for their child (*n* = 1865, 87.81%). Children had a mean age of 11.26 years (s.d. = 3.61), most were full-time students (*n* = 1940, 91.29%) and only a minority were part-time students (*n* = 143, 6.73%) or had a job (*n* = 29, 1.36%); 5.8% of parents (*n* = 123) had a child older than 18 years, and 9.8% of those above 18 (*n* = 12) reported they were working. Only a small proportion of children had a physical (*n* = 90, 4.24%) or mental health condition (*n* = 132, 6.21%).

**Table 1:** Participants’ demographic characteristics. Data expressed as total number, percentage, mean and s.d

Individual variables	*n*	%	Mean	s.d.
Parent’s age	2124		36.77	5.84
Gender				
Male	1067	50.24		
Female	1037	48.82		
Other	20	0.94		
Ethnicity				
African	1304	61.39		
Caribbean	303	14.27		
Black Other	15	0.71		
African-White	299	14.07		
African-Asian	46	2.17		
African-Latin American	5	0.24		
Caribbean-White	137	6.45		
Caribbean-Asian	12	0.56		
Caribbean Latin American	3	0.14		
Relationship status				
Married/in a civil partnership	1948	91.72		
Separated, but still legally married	51	2.41		
Divorced	57	2.69		
Widowed	18	0.85		
Single	50	2.35		
Employment status				
Full-time work	1566	73.73		
Part-time work	483	22.74		
Casual work	48	2.26		
Student	14	0.66		
Student + work	6	0.28		
Unemployed	5	0.23		
Retired	2	0.09		
Home status				
Owned	1530	72.03		
Rented	572	26.93		
Accommodation comes with the job	19	0.89		
Home facilities				
Access to private garden	720	33.90		
Access to communal garden	1095	51.55		
No access to garden	309	14.55		
No. of children				
1	1582	74.45		
2	412	19.40		
3	104	4.90		
4+	27	1.25		
Primary caregiver				
Yes	1865	87.81		
No	259	12.19		
Physical illness				
Yes	229	10.80		
No	1892	89.20		
Mental illness				
Yes	222	10.47		
No	1899	89.53		
Child age (6–24 years)			11.26	3.61
Child 18–24 years old	123	5.8		
Child education/employment status				
Full-time student	1940	91.29		
Part-time student	143	6.73		
Full-time work	13	0.61		
Part-time work	16	0.75		
Other	12	0.56		
Child physical illness				
Yes	90	4.24		
No	2034	95.76		
Child mental illness				
Yes	132	6.21		
No	1992	93.79		

### Association between parental well-being and children’s difficulties

**Fig. 1:**
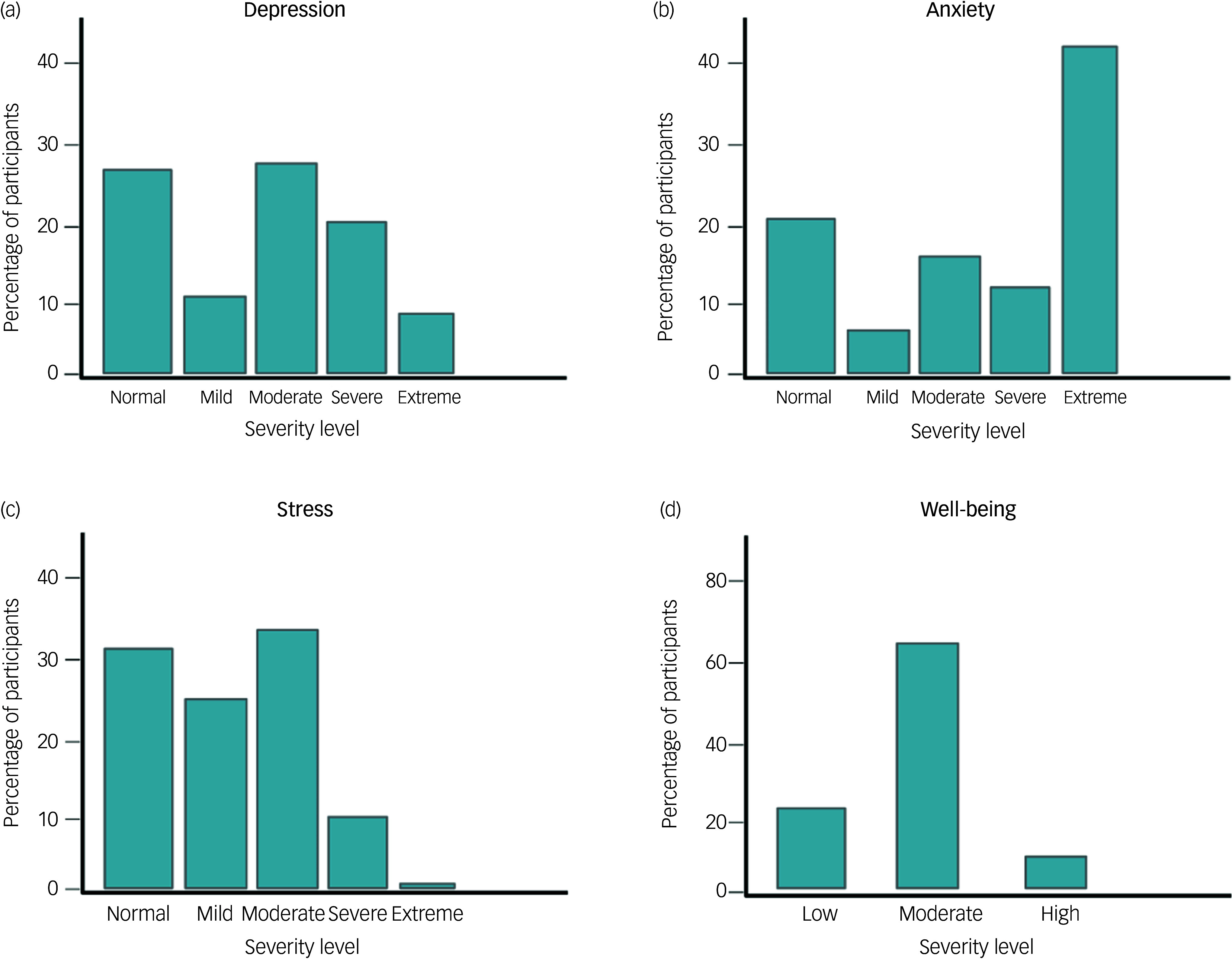
Distribution of parents’ responses based on symptom severity categories for (a) depressive symptoms, (b) anxiety symptoms, (c) stress symptoms of the Depression Anxiety and Stress Scale (DASS-21) and (d) well-being levels of the Warwick-Edinburgh Mental Well-being Scale (WEMWBS).

Parents completed the DASS-21 to measure symptoms of depression, anxiety and stress, and the WEMWBS to assess overall psychological well-being. The mean score for parents’ depressive symptoms was 15.42 (s.d. = 9.09). Almost half of the sample fell within the cut-off for mild to moderate depressive symptoms, 30.91% reported severe to extremely severe symptoms and only 28.44% scored within the ‘normal’ range (Fig. 1(a)). The mean score for anxiety symptoms was 15.72 (s.d. = 9.13). The greatest proportion scored in the severe to extremely severe anxiety range (55.25%) (Fig. 1(b)). Only one fifth scored within the normal range (21.11%) or within the cut-off for mild to moderate anxiety (23.64%). The mean score for stress levels was 16.39 (s.d. = 8.82). Approximately a third (30.67%) had scores within the normal range, whereas higher proportions reported mild to moderate (58.2%) or severe to extremely severe stress levels (11.13%) (Fig. 1(c)). The mean psychological well-being score on WEMWBS was 47.57 (s.d. = 8.50), with 23.89% reporting low levels of well-being, 66.27% reporting scores within the normal range (between 42 and 60) and 9.83% reporting high levels of well-being (Fig. 1(d)). Half of the sample estimated high levels of difficulties in their offspring in the SDQ (*n* = 1211, 57.66%; mean 16.43, s.d. = 6.54).

### Parental support and healthcare needs during COVID-19

**Fig. 2:**
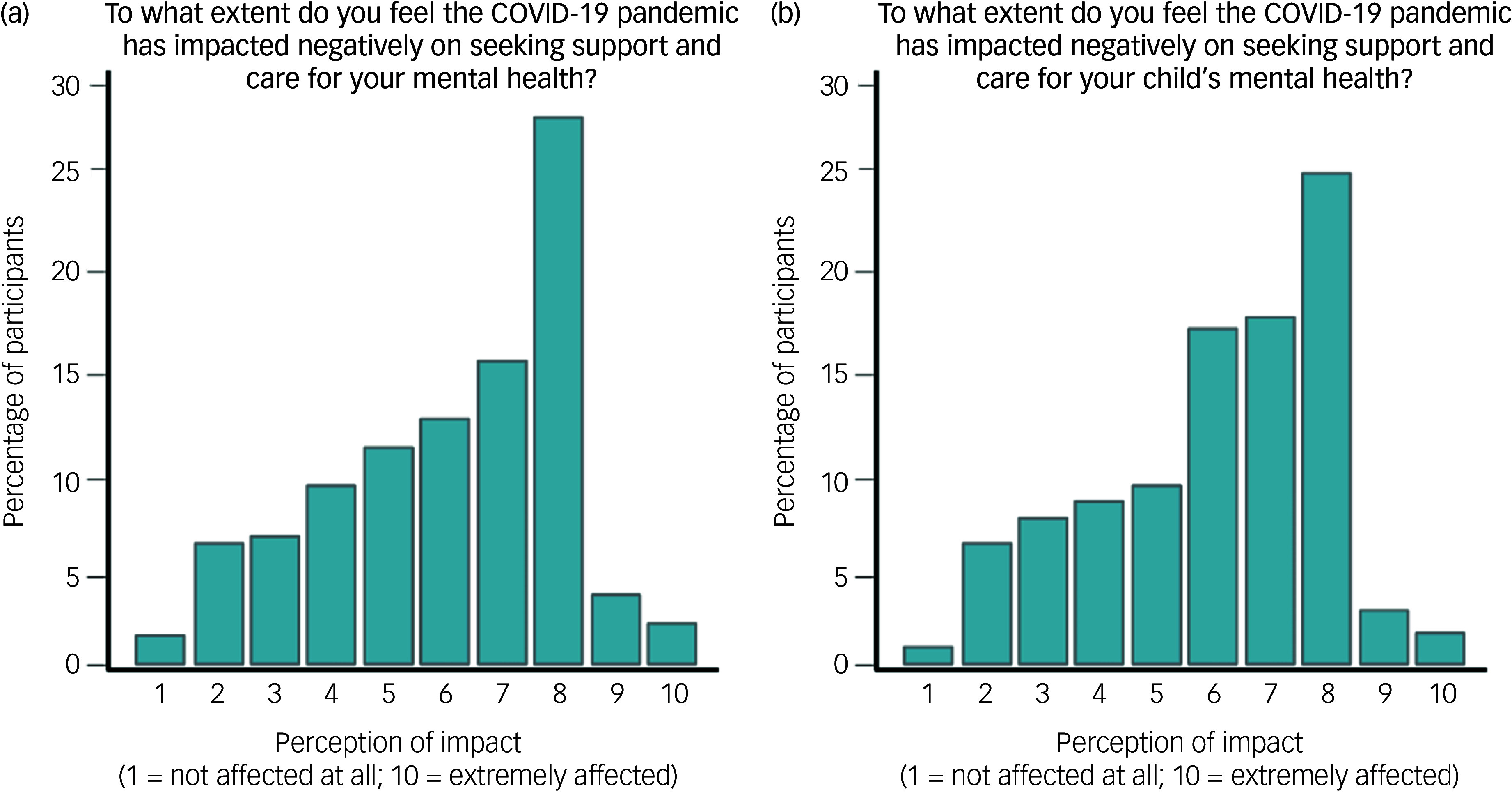
Distribution of parents’ responses regarding the negative impact of the COVID-19 pandemic on seeking support for (a) own mental health and (b) child’s mental health. Scores are on a scale from 1 (’not affected’) to 10 (’extremely affected’).

Since the beginning of the pandemic, approximately a third of the respondents had had to seek support for their own physical (30.98%) or mental health (34.56%), and about 40% had had to seek support for the physical (38.79%) or mental (43.66%) health of their children (Fig. 2). However, the negative impact of the pandemic on their’s and their child’s physical or mental health (including feeling exhausted in the caregiving role) was rated as ‘average’ (median 5 on Likert scale ranging from 0 to 10). Slightly higher scores were given to the negative impact of the pandemic on the ability to seek support for their’s or their child’s physical or mental health (median 6 and 7, respectively) (Table 2).

**Table 2:** Descriptive statistics for the impact of the COVID-19 pandemic on parents and their children

Individual variables	*n*	%	Mean	Median	s.d.
Support sought for child’s physical health					
Yes	824	38.79			
No	1300	61.21			
Support sought for child’s mental health					
Yes	927	43.66			
No	1196	56.34			
Support sought for own physical health					
Yes	657	30.98			
No	1464	69.02			
Support sought for own mental health					
Yes	733	34.56			
No	1388	65.44			
Negative impact of the pandemic on (1–10):					
Child’s relationships			5.30	5	2.24
Child’s physical health			4.94	5	2.26
Child’s mental health			5.06	5	2.25
Support sought for child’s physical health			5.81	6	2.04
Support sought for child’s mental health			6.02	6	2.07
Negative impact of the pandemic on (1–10):					
Relationship with children			5.24	5	2.46
Parents’ physical health			5.02	5	2.28
Parents’ mental health			5.03	5	2.26
Support sought for own physical health			5.67	6	2.10
Support sought for own mental health			6.09	7	2.18
Emotional/social support			5.06	5	2.21
Parental role after COVID-19 (1–10):					
Feeling physically exhausted			5.18	5	2.18
Feeling mentally exhausted			5.10	5	2.20

Respondents did not perceive significant changes in the extent to which they felt supported in their parental role by family, school, friends, their GP and the ethnic community since the pandemic, compared with the preceding period (Table 3).

**Table 3:** Perceived support in parental role pre- to post-COVID-19

Extent to which you feel supported in your parental role by (scale 1–5)	Pre-COVID-19 mean (s.d.)	Post-COVID-19 mean (s.d.)	*t* (*P*)
Family	3.18 (1.05)	3.19 (1.09)	−0.49 (0.622)
School	2.87 (1.11)	2.86 (1.09)	0.38 (0.708)
Friends	3.07 (1.02)	3.07 (1.01)	0.17 (0.865)
General practitioner	2.90 (1.07)	2.94 (1.05)	−1.46 (0.146)
Ethnic community	2.94 (1.01)	2.96 (1.03)	−0.99 (0.324)

### Association between parents’ well-being, likelihood of seeking support and children’s well-being

**Fig. 3:**
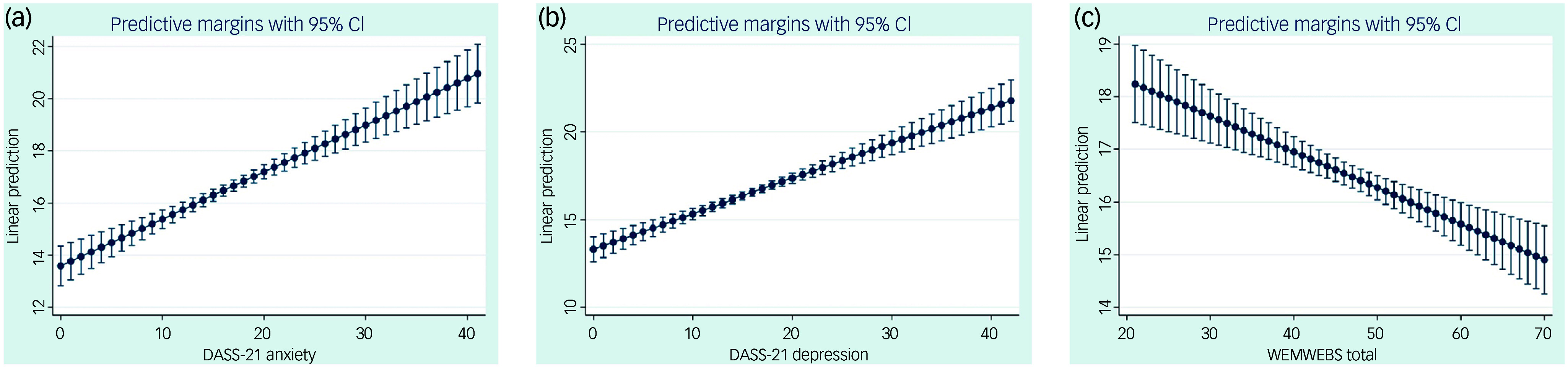
Associations between difficulties perceived in children (rated on the Strengths and Difficulties Questionnaire, SDQ) and parents’ (a) anxiety scores, (b) depression scores (DASS-21) and (c) well-being scores (WEMWBS). DASS-21, Depression, Anxiety and Stress Scale; WEMWBS, Warwick-Edinburgh Mental Well-being Scale.

The first set of regression assessed whether parents’ physical and psychological health were associated with their child’s behavioural and emotional difficulties (model 1), and their likelihood of seeking support for the child’s mental health during the pandemic (model 2). The results of these models are reported in Table 4. Variables related to parents’ psychological and mental well-being explained about 41% of the variance in children’s difficulties (total scores for SDQ, model 1). Most predictors exerted a significant effect. Higher symptoms of depression, anxiety and stress in parents were associated with greater difficulties in children, as shown in Fig. 3(a) and (b). Higher levels of parental well-being were associated with fewer children’s difficulties (Fig. 3(c)). Positive associations were observed between parental physical exhaustion and the presence of a physical illness and children’s difficulties. No effects were found for mental exhaustion or mental illness.

**Table 4:** Impact of parents’ levels of anxiety, depression and stress symptoms and mental health well-being on their children’s difficulties and on their ability to seek support for their children’s mental health

Parental physical and mental health variables	Model 1	Model 2
	Perceived difficulties in the child (SDQ)	Seeking support for child’s mental health
Anxiety symptoms (DASS-21)	0.18***	0.01
	(0.02)	(0.01)
Depression symptoms (DASS-21)	0.20***	−0.01
	(0.02)	(0.01)
Stress symptoms (DASS-21)	0.05**	0.01
	(0.02)	(0.01)
Mental well-being (WEMWBS)	−0.07***	0.01
	(0.01)	(0.01)
Parental physical exhaustion	0.22***	0.10***
	(0.06)	(0.03)
Parental mental exhaustion	−0.09	0.09***
	(0.06)	(0.03)
Parental physical illness	0.99***	0.56***
	(0.34)	(0.16)
Parental mental illness	0.33	2.25***
	(0.44)	(0.30)
Constant	10.69***	−4.72***
	(0.96)	(0.48)
Observations	2100	2103
*R* ^2^/pseudo-*R* ^2^	0.41	0.07

Robust standard errors in parentheses. SDQ, Strength and Difficulties Questionnaire; DASS-21, Depression, Anxiety and Stress Scale; WEMWBS, Warwick-Edinburgh Mental Well-being Scale.

***P < 0.01, **P < 0.05, *P < 0.1.

Although the fit for model 2 was worse compared with model 1, two important elements emerged. First, no significant correlations were found between DASS-21 subscales or WEMWBS scores and the likelihood of seeking support for children’s mental health. The only variables associated with a greater likelihood of seeking support were parents’ physical and mental exhaustion and a physical or mental illness. A parent with a physical illness was 12% more likely to seek support for their child’s mental health than one with no physical illness. A parent with a mental illness was more than twice as likely to seek support for their child’s mental health than one with no mental illness (40 *v*. 85%).

### Association between perceived support, parental psychological health and children’s difficulties

The second set of regressions examined a potential association between parents’ perceptions of support from family, school, friends, their GP and the ethnic community following COVID-19 and their own well-being (as measured by WEMWBS and DASS-21), or their children’s difficulties (measured by SDQ). The results of the models are reported in Table 5. Model 1 focused on parental well-being, models 2, 3 and 4 on parental levels of depression, anxiety and stress symptoms, respectively, and model 5 explored the association of these variables with perceived difficulties in children. Higher perception of support from family, friends and the community was associated with higher levels of parental well-being, whereas no associations were found with support from school and their GP. Parents feeling that the pandemic had had little impact on their capacity to seek emotional support experienced higher levels of well-being than those who felt that COVID-19 had impacted negatively on their abilities to seek help. Models 2, 3 and 4 explained the impact of the predictors on the DASS-21 subscales and demonstrated similar predictive power, with *R* ^2^ values slightly below 20%. These models consistently indicated that support from family and friends was associated with better mental health outcomes. Decreased perceived support from family and friends was linked to higher levels of anxiety, stress and depression. Community support was associated with lower stress but did not have a significant association with anxiety or depression. Across all models, feeling supported by the school was associated with higher levels of anxiety, stress and depression. However, these effects were small in magnitude (about half of the size of the effects of family support). Feeling supported by a GP was also associated with greater levels of anxiety and depression, while it had no impact on stress. Those who felt negatively affected by the pandemic in regard to their capacity to seek support scored high on all three indicators of poor mental health, and perceived more difficulties in the child. As shown by model 5, higher perceived support from family and the community was associated with fewer difficulties in the offspring, while both school and GP support were associated with greater difficulties (i.e. seeking support from schools or GPs might be associated with experiencing higher levels of difficulties).

**Table 5:** Parents’ perception of support from family, school, friends, general practitioner (GP) and the ethnic community following COVID-19, and impact on their psychological health and children’s difficulties

Variables	Model 1: parental mental well-being	Model 2: parental stress symptoms	Model 3: parental anxiety symptoms	Model 4: parental depressive symptoms	Model 5: perceived difficulties in the child
Perceived support from family	1.06***	−1.39***	−1.52***	−1.58***	−0.95***
	(0.17)	(0.18)	(0.18)	(0.18)	(0.13)
Perceived support from school	0.08	0.66***	0.37*	0.64***	0.72***
	(0.18)	(0.18)	(0.19)	(0.18)	(0.14)
Perceived support from friends	1.13***	−0.91***	−0.98***	−0.85***	−0.90***
	(0.20)	(0.20)	(0.21)	(0.20)	(0.15)
Perceived support from GP	0.30	0.14	0.63***	0.38**	0.28*
	(0.19)	(0.18)	(0.20)	(0.19)	(0.15)
Perceived support from the community	0.55***	−0.38*	0.01	−0.23	0.04
	(0.19)	(0.20)	(0.21)	(0.20)	(0.15)
Perception that the COVID-19 pandemic affected the ability to seek support	−0.26***	0.72***	0.62***	0.59***	0.36***
	(0.10)	(0.10)	(0.10)	(0.10)	(0.07)
Perception that the COVID-19 pandemic affected the relationship with the child	−0.15**	0.29***	0.28***	0.32***	0.32***
	(0.07)	(0.08)	(0.08)	(0.08)	(0.06)
Perception that the COVID-19 pandemic affected the child’s relationship skills	−0.37***	0.77***	0.86***	0.90***	0.42***
	(0.10)	(0.10)	(0.10)	(0.10)	(0.07)
Constant	42.06*** (1.17)	13.23*** (1.03)	11.55*** (1.07)	11.39*** (1.06)	13.44*** (0.76)
Observations	2105	2103	2103	2103	2100
*R* ^2^	0.11	0.19	0.17	0.18	0.14

Robust standard errors in parentheses.

***P < 0.01, **P < 0.05, *P < 0.1.

### Association between children’s difficulties and impact of COVID-19 on parent–child relationship and parental psychological health

Parents reporting that the relationship with their children had not been affected, or only minimally affected, by the pandemic experienced higher levels of well-being, whereas the perception that the pandemic had had a stronger impact on the parent–child relationship was associated with more perceived difficulties in the child.

### Association between children’s difficulties and the impact of COVID-19 on children’s relational abilities and parental psychological health

Parents of children whose interpersonal abilities had not suffered from the impact of the pandemic scored about three points higher in WEBWBS than those whose children’s relationships had been extremely disrupted. Also, parents who reported an extreme deterioration in their children relational capacity following the pandemic felt eight points more stressed and nine points more depressed and anxious than those whose offspring experienced no effect of COVID-19 on their relational capacities. The perception that COVID-19 had affected the child’s relationship skills was also associated with greater perceived difficulties in the child.

## Discussion

Despite exposure to several stress factors, literature on the psychological and emotional burden faced by individuals from a Black ethnic background is scarce.^21^ The present study contributed to this literature by assessing the healthcare needs of more than 2000 parents from communities with a Black ethnic background in the UK, soon after the COVID-19 pandemic. Over 70% of participants scored above the ‘normal’ range on measures of depression, anxiety and stress symptoms. This proportion is much higher than the prevalence of symptoms of depression, anxiety and stress reported in a recent synthesis of the literature on the mental health effects of the COVID-19 pandemic in different countries (i.e. 37.0, 31.0 and 29.6% for depression, anxiety and stress, respectively^22^). However, in striking contrast with the high proportion of people experiencing symptoms of anxiety, depression and stress in this study, only approximately 10% had a diagnosed mental health condition and only a third reported seeking assistance for their own physical and mental health. A similar picture emerged with regard to children’s diagnosed health conditions, reported by approximately 5% of parents, despite the high prevalence of significant difficulties observed (almost 60% of parents rated high levels of difficulties in their children). These data seem to indicate that parents face difficulties in seeking professional support, despite the recognition of psychological difficulties in themselves or their children.^23,24^

The endured challenges faced by individuals from a Black ethnic background could also explain the finding that parents did not feel that the pandemic had had a significant impact on the overall well-being of their family, or the extent to which they felt supported by institutions and services, the community and significant others after the pandemic, compared with previously. However, this hypothesis remains to be tested with adequate research designs. Also, it is of note that parents experiencing a greater negative impact of the pandemic on their ability to seek support for their parental role also reported greater difficulties in their children. This might suggest that difficulties in accessing care have a deleterious impact on children’s psychological well-being. On the other hand, feeling supported by members of the close community was associated with greater well-being in parents and with fewer perceived difficulties in their children. Supportive environments can promote positive parenting and improve children’s psychological adjustment.^25–27^ However, reliance on informal support networks might result in insufficient help for parents who experience severe psychological difficulties.^28–30^ This hypothesis seems plausible considering that, in this study, the level of support received from formal networks, such as the GP or schools, was associated with high levels of anxiety and depression in parents.

When considering parents’ perception of their children’s needs and well-being, it became apparent that a greater proportion of parents sought support for their children’s difficulties (over half) compared with those seeking support for their own problems (a third). Feelings of exhaustion in the caregiving role represented an important determinant of the choice to seek support for their children. Parents’ responsiveness to children’s difficulties was also indicated by the strong relationships between indicators of parents’ mental health problems (i.e. symptoms of anxiety, depression and stress) and children’s difficulties, including problems in the interpersonal domain, and between children’s difficulties and parents’ feelings of exhaustion. Consistent with previous literature,^31–33^ also in this study parents’ and children’s well-being were strongly mutually associated.

Overall, data seem to suggest that healthcare access is still suboptimal in communities with a Black ethnic background, despite the high levels of mental health difficulties experienced within families. Together with political and societal efforts to reduce barriers to care and improve equity and inclusivity in clinical services, mental health professionals should focus on the implementation of culturally sensitive and personalised approaches to parents’ and children’s well-being. This process starts from a collaborative approach with members of ethnic minority groups to identify needs, priorities, resources, implementation and evaluation systems that consider individuals’ preferences and values. An example of such approach is the adaptation of evidence-based parenting programmes, such as the Positive Parenting Programme (Triple P), to improve parents’ knowledge, skills and confidence to promote children’s well-being, to refugees^34^ or the use of parenting programmes culturally adapted for implementation in African countries.^35^

### Limitations

Despite the use of community-based and media recruitment methods to maximise representation and inclusion (i.e. at the potential expense of statistical generalisability^36^), participation in the study was biased by specific characteristics, such as ethnic background and socioeconomic status. Most participants reported having a Black African ethnic background, and therefore there was a smaller representation of people with a mixed heritage background, who tend to experience even greater mental health challenges and barriers to care.^23,24^ Similarly, approximately 70% of participants were in full-time employment and reported owning a house. This percentage appears higher than those documented in UK national statistics (e.g. around 50% of people with a Black ethnic background or mixed Black ethnic background reported an ‘employee status’ in the 2021 Census^37^), and might suggest that those willing to participate in research were in more favourable socioeconomic circumstances. Recruitment bias might have been introduced also by the request to access a mobile device and the internet to complete the survey. This was done to ensure anonymity, but might have limited participation due to the digital divide. Even though the findings may not be representative of Black ethnic minorities in the UK, we feel it is important to document the views of those who chose to participate. This is a small step towards the inclusion of people from under-represented groups in research, despite recruitment and sampling issues. Dismissing these findings because of the suboptimal level of representativeness might concur with keeping minorities excluded from the research process. Also, the involvement of some group members (even those with a higher socioeconomic status) might help with reaching out to those with a less favourable socioeconomic status. The challenge to broaden inclusion and representativeness of people from ethnic minority groups in research remains, and could require the use of different and novel strategies to produce satisfactory outcomes.^38^

A second important point to consider is that the absence of a control group from other ethnic backgrounds might have limited the interpretation and comparison of the data in this study. The decision to focus on people from a Black ethnic background was shaped by consultations with the Funder and the study advisory group, and was based on the resources allocated to the project (i.e. budget available, timeline for data collection), expected barriers to recruitment (e.g. stigma and mistrust) and the scope of the call (focusing on one specific group to enable clearer generalisability of findings.^39^

Finally, a decision was made to ask carers to focus on one of their children (i.e. on the child experiencing greatest difficulties). This might have limited the understanding of the broader life circumstances and well-being of the families involved.

High rates of psychological distress have been documented among families with a Black ethnic background. The COVID-19 pandemic may have exacerbated difficulties in accessing care and receiving support in a subgroup of individuals, while most participants felt that the pandemic did not have a marked impact on families’ well-being. This might be due to the continued exposure to stressors endured by Black ethnic communities even prior to the COVID-19 pandemic. Psychological difficulties were mitigated, to an extent, by informal networks of support, with people experiencing greater mental health difficulties recognising the need to seek support from more formal networks, such as their GP or schools. Parents’ and children’s well-being levels were highly inter-related, suggesting that interventions targeting the family system might result in significant positive changes.

## Supporting information

Cardi et al. supplementary material Cardi et al. supplementary material

## Data Availability

The data that support the findings of this study are available from the corresponding author, V.C., upon reasonable request.

## Supplementary material

The supplementary material can be found online at https://doi.org/10.1192/bjo.2025.10049

## References

[1] Ali DA , Figley CR , Tedeschi RG , Galarneau D , Amara S. Shared trauma, resilience, and growth: a roadmap toward transcultural conceptualization. Psychol Trauma 2021; 15: 45–55. 34138612 10.1037/tra0001044

[2] Fullerton CS , Ursano RJ. Psychological and psychopathological consequences of disasters. In Disasters and Mental Health (eds JJ López-Ibor , G Christodoulou , M Maj , N Sartorius , A Okasha ): 13–36. John Wiley & Sons Ltd, 2005.

[3] Bourmistrova NW , Solomon T , Braude P , Strawbridge R , Carter B. Long-term effects of COVID-19 on mental health: a systematic review. J Affect Disord 2022; 299: 118–25. 34798148 10.1016/j.jad.2021.11.031 PMC8758130

[4] Collantoni E , Saieva AM , Meregalli V , Girotto C , Carretta G , Boemo DG , et al. Psychological distress, fear of covid-19, and resilient coping abilities among healthcare workers in a tertiary first-line hospital during the coronavirus pandemic. J Clin Med 2021; 10: 1465. 33918169 10.3390/jcm10071465 PMC8038142

[5] Tai DBG , Shah A , Doubeni CA , Sia IG , Wieland ML. The disproportionate impact of COVID-19 on racial and ethnic minorities in the United States. Clin Infect Dis 2021; 72: 703–6. 32562416 10.1093/cid/ciaa815 PMC7337626

[6] Agyemang C , Richters A , Jolani S , Hendriks S , Zalpuri S , Yu E , et al. Ethnic minority status as social determinant for COVID-19 infection, hospitalisation, severity, ICU admission and deaths in the early phase of the pandemic: a meta-analysis. BMJ Glob Health 2021; 6: e007433. 10.1136/bmjgh-2021-007433 PMC8573300 34740916

[7] Gupta A , Bernard C , Lakhanpaul M , Sharma A , Peres T , Schack L . *The Consortium on Practices of Wellbeing and Resilience in BAME Families and Communities: Children, Young People and their Families. Project Report*. UCL/Goldsmiths, University of London/Royal Holloway, University of London, 2023 (https://research.gold.ac.uk/id/eprint/33712/13/Consortium%20on%20Practices%20of%20Wellbeing%20Report%20WEB%20v2.pdf).

[8] Razai MS , Osama T , McKechnie DGJ , Majeed A. Covid-19 vaccine hesitancy among ethnic minority groups. BMJ 2021; 372: n513. 33637577 10.1136/bmj.n513

[9] Gur RE , White LK , Waller R , Barzilay R , Moore TM , Kornfield S , et al. The disproportionate burden of the COVID-19 pandemic among pregnant black women. Psychiatry Res 2020; 293: 113475. 33007683 10.1016/j.psychres.2020.113475 PMC7513921

[10] Nguyen LH , Anyane-Yeboa A , Klaser K , Merino J , Drew DA , Ma W , et al. The mental health burden of racial and ethnic minorities during the COVID-19 pandemic. PLoS ONE 2022; 17: e0271661. 35947543 10.1371/journal.pone.0271661 PMC9365178

[11] Thomeer M , Moody M , Yahirun J. Racial and ethnic disparities in mental health and mental health care during the COVID-19 pandemic. J Racial Ethn Health Disparities 2022; 10: 961–76. 35318615 10.1007/s40615-022-01284-9 PMC8939391

[12] Miconi D , Rousseau C , Frounfelker R , Li Z , Santavicca T , Cénat JM , et al. Ethno-cultural disparities in mental health during the COVID-19 pandemic: a cross-sectional study on the impact of exposure to the virus and COVID-19-related discrimination and stigma on mental health across ethno-cultural groups in Quebec (Canada). BJPsych Open 2020; 7: e14. 33295270 10.1192/bjo.2020.146 PMC7844156

[13] Siegel M , Critchfield-Jain I , Boykin M , Owens A. Actual racial/ethnic disparities in COVID-19 mortality for the non-hispanic black compared to non-hispanic white population in 35 US states and their association with structural racism. J Racial Ethn Health Disparities 2022; 9: 886–98. 33905110 10.1007/s40615-021-01028-1 PMC8077854

[14] Best A , Fletcher F , Kadono M , Warren R. Institutional distrust among African Americans and building trustworthiness in the COVID-19 response: implications for ethical public health practice. J Health Care Poor Underserved 2021; 32: 90–8. 33678683 10.1353/hpu.2021.0010 PMC7988507

[15] Collaborating Centre for Mental Health and NHS Race & Health Observatory. *Ethnic Inequalities in Improving Access to Psychological Therapies (IAPT) Full Report*. Collaborating Centre for Mental Health and NHS Race & Health Observatory, 2023 (https://www.nhsrho.org/wp-content/uploads/2023/10/Ethnic-Inequalities-in-Improving-Access-to-Psychological-Therapies-IAPT.Full-report.pdf).

[16] Henry JD , Crawford JR. The short-form version of the Depression Anxiety Stress Scales (DASS-21): construct validity and normative data in a large non-clinical sample. Br J Clin Psychol 2005; 44: 227–39. 16004657 10.1348/014466505X29657

[17] Tennant R , Hiller L , Fishwick R , Platt S , Joseph S , Weich S , et al. The Warwick-Dinburgh mental well-being scale (WEMWBS): development and UK validation. Health Qual Life Outcomes 2007; 5: 63. 18042300 10.1186/1477-7525-5-63 PMC2222612

[18] Brann P , Lethbridge MJ , Mildred H. The young adult Strengths and Difficulties Questionnaire (SDQ) in routine clinical practice. Psychiatry Res 2018; 264: 340–5. 29674224 10.1016/j.psychres.2018.03.001

[19] Goodman R. The strengths and difficulties questionnaire: a research note. J Child Psychol Psychiatry 1997; 38: 581–6. 9255702 10.1111/j.1469-7610.1997.tb01545.x

[20] Lovibond PF , Lovibond SH. The structure of negative emotional states: comparison of the depression anxiety stress scales (DASS) with the beck depression and anxiety inventories. Behav Res Ther 1995; 33: 335–43. 7726811 10.1016/0005-7967(94)00075-u

[21] Goodwill JR. Purpose in the pandemic: fear of COVID-19, hopelessness, meaning in life, and suicidal thoughts among two samples of Black Americans. Am Psychol 2023; 78: 775–89. 37428777 10.1037/amp0001171

[22] Salari N , Hosseinian-Far A , Jalali R , Vaisi-Raygani A , Rasoulpoor S , Mohammadi M , et al. Prevalence of stress, anxiety, depression among the general population during the COVID-19 pandemic: a systematic review and meta-analysis. Glob Health 2020; 16: 57. 10.1186/s12992-020-00589-w PMC7338126 32631403

[23] Alam S , O’Halloran S , Fowke A. What are the barriers to mental health support for racially-minoritised people within the UK? A systematic review and thematic synthesis. Cogn Behav Ther 2024; 17: e10.

[24] Bornheimer LA , Acri MC , Gopalan G , McKay MM. Barriers to service utilization and child mental health treatment attendance among poverty-affected families. Psychiatr Serv 2018; 69: 1101–4. 29983111 10.1176/appi.ps.201700317 PMC6167148

[25] Hosokawa R , Katsura T. Association between parents’ perceived social support and children’s psychological adjustment: a cross-sectional study. BMC Pediatr 2024; 24: 756. 39567953 10.1186/s12887-024-05235-7 PMC11580202

[26] Brown SM , Doom JR , Lechuga-Peña S , Watamura SE , Koppels T. Stress and parenting during the global COVID-19 pandemic. Child Abuse Negl 2020; 110: 104699. 32859394 10.1016/j.chiabu.2020.104699 PMC7440155

[27] Lee SJ , Ward KP , Lee JY , Rodriguez CM. Parental social isolation and child maltreatment risk during the COVID-19 pandemic. J Fam Violence 2022; 37: 813–24. 33462526 10.1007/s10896-020-00244-3 PMC7807402

[28] Lin S (Lamson). The ‘loneliness epidemic’, intersecting risk factors and relations to mental health help-seeking: a population-based study during COVID-19 lockdown in Canada. J Affect Disord 2023; 320: 7–17. 36058359 10.1016/j.jad.2022.08.131 PMC9436782

[29] Heron P , Spanakis P , Crosland S , Johnston G , Newbronner E , Wadman R , et al. Loneliness among people with severe mental illness during the COVID-19 pandemic: results from a linked UK population cohort study. PLoS ONE 2022; 17: e0262363. 35025915 10.1371/journal.pone.0262363 PMC8757957

[30] Schickedanz A , Halfon N , Sastry N , Chung PJ. Parents’ adverse childhood experiences and their children’s behavioral health problems. Pediatrics 2018; 142: e20180023. 29987168 10.1542/peds.2018-0023 PMC6317990

[31] Neece CL , Green SA , Baker BL. Parenting stress and child behavior problems: a transactional relationship across time. Am J Intellect Dev Disabil 2012; 117: 48–66. 22264112 10.1352/1944-7558-117.1.48 PMC4861150

[32] Guajardo NR , Snyder G , Petersen R. Relationships among parenting practices, parental stress, child behaviour, and children’s social-cognitive development. Infant Child Dev 2009; 18: 37–60.

[33] Poppert Cordts KM , Wilson AC , Riley AR. More than mental health: parent physical health and early childhood behavior problems. J Dev Behav Pediatr 2020; 41: 265–71. 31688659 10.1097/DBP.0000000000000755 PMC7190438

[34] Zagha K , Konietzny K , Brettschneider C , Chehadi O , Chehadi-Köster A , Chikhradze N , et al. Improve Mental Health (Improve-MH) in refugee families using a culturally adapted, general practitioner-delivered psychotherapeutic intervention combined with Triple P Online parenting programme: study protocol of a multicentre randomised controlled trial. BMJ Open 2024; 14: e084080. 10.1136/bmjopen-2024-084080 PMC11423745 39317509

[35] Asiimwe R , Dwanyen L , Subramaniam S , Kasujja R , Blow AJ. Training of interventionists and cultural adaptation procedures: a systematic review of culturally adapted evidence-based parenting programs in Africa. Fam Process 2023; 62: 160–81. 35570371 10.1111/famp.12780

[36] Harris KJ , Ahluwalia JS , Catley D , Okuyemi KS , Mayo MS , Resnicow K. Successful recruitment of minorities into clinical trials: the Kick It at Swope project. Nicotine Tobacco Res 2003; 5: 575–84. 10.1080/1462220031000118540 12959796

[37] Office for National Statistics (ONS). *ONS Website, Statistical Bulletin, Ethnic group, England and Wales: Census 2021*. ONS, 2022 (https://www.ons.gov.uk/peoplepopulationandcommunity/culturalidentity/ethnicity/bulletins/ethnicgroupenglandandwales/census2021).

[38] Marpsat M , Razafindratsima N. Survey methods for hard-to-reach populations: introduction to the special issue. Methodol Innov Online 2010; 5: 3–16.

[39] Jager J , Putnick DL , Bornstein MH II . More than just convenient: the scientific merits of homogeneous convenience samples. Monogr Soc Res Child Dev 2017; 82: 13–30. 28475254 10.1111/mono.12296 PMC5606225

