# Supplementary material for: The impact of COVID-19 on parents from Black ethnic backgrounds in the UK: what we have learned and why it still matters
Source: BJPsych Open. 2025 Jul 8;11(4):e143. doi: 10.1192/bjo.2025.10049 (PMC12247070; doi:10.1192/bjo.2025.10049)
Supplement: Cardi et al. supplementary material [file S2056472425100495sup001.docx]

**Supplementary Materials**

***Supplementary Materials.*** Integral version of the COVID-19 Survey.

| **VARIABLE** | **VALUE** |
| --- | --- |
| QUESTIONS RELATED TO THE RESPONDENT (CAREGIVER) |  |
| Age |  |
| Gender (at birth) | 1 – Male  2 – Female  3 – Intersex  4 – Prefer not to say |
| Gender (identity) | 1 – Male  2 – Female  3 - Other |
| For gender, if other, please specify: | Open text |
| What is your ethnic group?  Consider only the last column: ETHNICITY | African  Caribbean  Mixed African-White  Mixed African-Asian  Mixed African-Latin American  Mixed African-Gypsy  Mixed African-American  Mixed Caribbean-white  Mixed Caribbean-Asian  Mixed Caribbean-Latin American |
| Relationship Status | 1 – Single, never married  2 – Married  3 – Separated but still legally married  4 – Divorced  5 – Widowed  6 – Registered civil partnership  7 – separated, still legally in civil partnership  8 – legally dissolved civil partnership  9 – surviving partner from civil partnership |
| Employment status | 1 – Full-time paid  2 – Part-time paid  3 – student  4 – retired  5 – casual work  6 – self-employed  7 – student with part time employment  8 – student with full time employment  9 – voluntary work  10 – other |
| If employment status is other, please specify: | Open text |
| Who do you live with? |  |
| Home status | 1 – owned, mortgage paid  2 – owned, still paying mortgage  3 – rented from local council/housing association  4 – rented from a private landlord  5 – accommodation comes with the job  6 – other |
| If your present home can be described by other, please specify: | Open text |
| N of rooms (Please do not count bathrooms, toilets, halls, or landings) | Open text |
| Home facilities | 1 – Access to private garden  2 – Access to communal garden  3 – Private and communal garden space  4 – No access to private or communal garden space |
| N of children (included adopted; please specify how many children you have, and live with you)? | Open text |
| Age children | Open text |
| Primary caregiver | 1 – yes  2 – no |
| QUESTIONS RELATED TO THE SPECIFIC CHILD |  |
| Age | Open text |
| Education/Employment status | 1 – Full-time student  2 – part-time student  3 – working full-time  4 – working part-time  5 – casual  6 – self-employed  7 – other |
| For your child's employment/education status, if other, please specify: | Open text |
| Physical illness | 1 – yes  2 – no |
| If yes, could you please tell us the name or more about the type of physical health problems experienced? | Open text |
| Mental health problems | 1 – yes  2 – no |
| If yes, could you please tell us the name or more about the type of mental health problems experienced? | Open text |
| Covid-relationships:  *“On the scale below, where 1 reflects not at all affected and 10 reflects extremely affected, please indicate to what extent COVID-19 has negatively affected the social relationships*  *of the child you care for:”* | 1 – not at all affected  2  3  4  5  6  7  8  9  10 – extremely affected |
| Covid-physical health:  *“On the scale below, where 1 reflects not at all affected and 10 reflects extremely affected, please indicate to what extent COVID-19 has negatively affected the physical health of the child you care for:”* | 1 – not at all affected  2  3  4  5  6  7  8  9  10 – extremely affected |
| Covid-mental health:  *“On the scale below, where 1 reflects not at all affected and 10 reflects extremely affected, please indicate to what extent COVID-19 has negatively affected the mental health of the child you care for:”* | 1 – not at all affected  2  3  4  5  6  7  8  9  10 – extremely affected |
| Covid Support physical:  *Since the start of COVID-19, have you had to seek support and care for your child’s physical health?* | 1 – yes  2 – no |
| Covid-support physical impact:  *If yes, on the scale below, where 1 reflects not at all affected and 10 reflects extremely affected, please indicate, to what extent you feel the COVID-19 pandemic has impacted negatively on seeking support and care for your child’s physical health:* | 1 – not at all affected  2  3  4  5  6  7  8  9  10 – extremely affected |
| Covid-support mental:  *Since the start of COVID-19, have you had to seek support and care for your child’s mental health?* | 1 – yes  2 – no |
| Covid-support mental impact  *If yes, on the scale below, where 1 reflects not at all affected and 10 reflects extremely affected, please indicate, to what extent you feel the pandemic has impacted negatively on seeking support and care for your child’s mental health:* | 1 – not at all affected  2  3  4  5  6  7  8  9  10 – extremely affected |
| Covid (more)  *Please tell us more about in what way, if any, COVID-19 has affected your experience of seeking support and care for your child:* | Open text |
| QUESTIONS RELATED TO RESPONDAND (CAREGIVER) |  |
| Physical illness | 1 – yes  2 – no |
| If yes, is there a name (e.g., diagnosis) that a professional has used to describe the problems that you experience? | Open text |
| Mental health problems | 1 – yes  2 – no |
| If yes, is there a name (e.g., diagnosis) that a professional has used to describe the problems that you experience? | Open text |
| Covid-physical health:  *On the scale below, where 1 reflects not at all affected and 10 reflects extremely affected, please indicate, to what extent COVID-19 has negatively affected your physical health:* | 1 – not at all affected  2  3  4  5  6  7  8  9  10 – extremely affected |
| Covid mental-health:  *On the scale below, where 1 reflects not at all affected and 10 reflects extremely affected, please indicate, to what extent COVID-19 has negatively affected your mental health:* | 1 – not at all affected  2  3  4  5  6  7  8  9  10 – extremely affected |
| Covid-support physical  *Since the start of COVID-19, have you had to seek support and care for your physical health?* | 1 – yes  2 – no |
| Covid-support physical impact:  *If yes, on the scale below, where 1 reflects not at all affected and 10 reflects extremely affected, please indicate, to what extent you feel the COVID-19 pandemic has impacted negatively on seeking support and care for your physical health:* | 1 – not at all affected  2  3  4  5  6  7  8  9  10 – extremely affected |
| Covid-support mental:  *Since the start of COVID-19, have you had to seek support and care for your mental health?* | 1 – yes  2 – no |
| Covid-support mental impact:  *If yes, on the scale below, where 1 reflects not at all affected and 10 reflects extremely affected, please indicate, to what extent you feel the COVID-19 pandemic has impacted negatively on seeking support and care for your mental health:* | 1 – not at all affected  2  3  4  5  6  7  8  9  10 – extremely affected |
| Covid (more)  *Please feel free to tell us more about what way, if any, COVID-19 has affected the care that you needed:* | Open text |
| QUESTIONS RELATED TO PARENTAL ROLE (PR) |  |
| PR1  On the scale below, where 1 reflects not at all affected and 10 reflects extremely affected, please indicate, to what extent you feel physically exhausted in your parental/ carer role: | 1 – not at all affected  2  3  4  5  6  7  8  9  10 – extremely affected |
| PR2  On the scale below, where 1 reflects not at all affected and 10 reflects extremely affected, please indicate, to what extent you feel mentally exhausted in your parental/ carer role: | 1 – not at all affected  2  3  4  5  6  7  8  9  10 – extremely affected |
| PR3  Do you have someone that you can rely on for emotional and social support? | 1 – yes  2 – no |
| PR4  Prior to COVID-19, to what extent did you feel supported in your parent/carer role, by your family: | 1 – not at all  2 – slightly  3 – moderately  4 – very  5 – extremely |
| PR5  Prior to COVID-19, to what extent did you feel supported in your parent/carer role, by your child's school: | 1 – not at all  2 – slightly  3 – moderately  4 – very  5 – extremely |
| PR6  Prior to COVID-19, to what extent did you feel supported in your parent/carer role, by your friends: | 1 – not at all  2 – slightly  3 – moderately  4 – very  5 – extremely |
| PR7  Prior to COVID-19, to what extent did you feel supported in your parent/carer role, by your GP: | 1 – not at all  2 – slightly  3 – moderately  4 – very  5 – extremely |
| PR8  Prior to COVID-19, to what extent did you feel supported in your parent/carer role, by members of your own ethnic community: | 1 – not at all  2 – slightly  3 – moderately  4 – very  5 – extremely |
| PR9  Since COVID-19, to what extent do you feel supported in your parent/carer role, by your family? | 1 – not at all  2 – slightly  3 – moderately  4 – very  5 – extremely |
| PR10  Since COVID-19, to what extent do you feel supported in your parent/carer role, by your child's school? | 1 – not at all  2 – slightly  3 – moderately  4 – very  5 – extremely |
| PR11  Since COVID-19, to what extent do you feel supported in your parent/carer role, by your friends? | 1 – not at all  2 – slightly  3 – moderately  4 – very  5 – extremely |
| PR12  Since COVID-19, to what extent do you feel supported in your parent/carer role, by your GP? | 1 – not at all  2 – slightly  3 – moderately  4 – very  5 – extremely |
| PR13  Since COVID-19, to what extent do you feel supported in your parent/carer role, by members of your own ethnic community? | 1 – not at all  2 – slightly  3 – moderately  4 – very  5 – extremely |
| PR14  Please list any other sources of support during COVID-19 that you have found helpful: | Open text |
| PR15  On the scale below, where 1 reflects not at all affected and 10 reflects extremely affected, please indicate, to what extent COVID-19 has affected your ability to seek emotional/social support (e.g., to speak to someone, etc) for yourself: | 1 – not at all affected  2  3  4  5  6  7  8  9  10 – extremely affected |
| PR16  If you wish to expand on your answer, please do so in the box below: | Open text |
| PR17  On the scale below, where 1 reflects not at all affected and 10 reflects extremely affected, to what extent COVID-19 has negatively affected your relationship with your children: | 1 – not at all affected  2  3  4  5  6  7  8  9  10 – extremely affected |
| PR18  Given your rating, can you please explain, in what way, if at all, COVID-19 has affected your relationship with your child: | Open text |
| PR19  In what way(s) might have others (e.g. schools/ family/ friends/ General Practitioners/ members of your own ethnic community improved the support you received for your child(ren) during COVID-19? | Open text |
| PR20  In what way(s) (if any at all) has COVID-19 affected your ability to seek help and support for your child’s difficulties? | Open text |
| PR21  Please use this section below to write down any comments that you have on areas that might not have been covered in the above questions or just how you have felt completing the above questions: | Open text |
